# Excretory-Secretory Products from Hookworm L_3_ and Adult Worms Suppress Proinflammatory Cytokines in Infected Individuals

**DOI:** 10.1155/2011/512154

**Published:** 2011-06-09

**Authors:** Stefan Michael Geiger, Ricardo Toshio Fujiwara, Paula Albuquerque Freitas, Cristiano Lara Massara, Omar dos Santos Carvalho, Rodrigo Corrêa-Oliveira, Jeffrey Michael Bethony

**Affiliations:** ^1^Centro de Pesquisas René Rachou, Fundação Oswaldo Cruz, Avenida Augusto de Lima 1715, 30190-002 Belo Horizonte, MG, Brazil; ^2^Departamento de Parasitologia, Instituto de Ciências Biológicas, Universidade Federal de Minas Gerais, Avenida Antônio Carlos 6627, 31270-901 Belo Horizonte, MG, Brazil; ^3^Department of Microbiology, Immunology, and Tropical Medicine, The George Washington University Medical Center, 2300 Eye Street NW, Washington, DC 20037, USA

## Abstract

We compared the effects of larval and adult worm excretory-secretory (ES) products from hookworm on the proliferative responses and cytokine secretion in peripheral blood mononuclear cells (PBMCs) from hookwormpatients and egg-negative, nonendemic controls. When compared with negative controls, mitogen-stimulated PBMC from hookworm-infected individuals showed a significantly reduced proliferative response when adult worm ES antigen was added to the cultures. Furthermore, in hookworm-infected individuals a significant downmodulation of inflammatory interleukin (IL)-6 and tumor necrosis factor (TNF)-*α* secretion resulted when PBMCs were stimulated with mitogen in combination with larval or adult worm ES. Both, interferon (IFN)-*γ* and IL-10 secretion were significantly lower in stimulated PBMC from infected individuals; however the IFN-*γ*/IL-10 ratio was much lower in hookworm-infected patients. Comparable effects, although at lower concentrations, were achieved when PBMCs from both groups were incubated with living hookworm third-stage larvae. We suggest that hookworm ES products downmodulate proliferative responses and inflammation during the chronic phase of the disease and facilitate early larval survival or adult worm persistence in the gut.

## 1. Introduction

Helminth excretory-secretory products (ES) contain a vast mixture of antigens, are potent modulators of the host's immune response, and therefore are important factors in worm survival and maintenance of a chronic human infection. ES products from different parasite species and different stages of parasitic development within the host were shown to downmodulate Type 1 immunity [1]. Among other mechanisms, early effects on dendritic cell function and innate immune responses have been previously described for intestinal nematode models [2], as well as for filarial infections [3, 4] with these events contributing to minimize inflammatory responses and induce a Type 2 response [1]. For hookworms, many components in ES products have been described so far [5, 6]; however, their effect on the human immune response is still not well understood and has become, more recently, a topic of intense investigation.

We have recently reported that PBMCs from *Necator americanus*-infected patients had a lower production of TNF-*α* and IL-10 in response to ES antigen derived from adult *Ancylostoma caninum. *Apart from the downmodulated cytokine secretion in response to ES antigen, PBMCs from endemic patients proliferated poorly in response to crude ES antigen extracts [7]. In the present study, we compare the effects of hookworm adult and larval ES antigen preparations on polyclonal-activated lymphocytes from two different groups—(1) individuals chronically infected with hookworm, residing in an area of high transmission and (2) egg-negative (“control”) and nonexposed individuals, residing in a nonendemic area. We found that proliferative responses and the secretion of proinflammatory cytokines in hookworm-infected individuals were downmodulated to a significant degree when compared to egg-negative controls.

## 2. Materials and Methods

### 2.1. Selection of Patients

In the present study, hookworm-infected adult individuals were recruited during an epidemiological field survey in 2006 in the village of Ladainha, located in the north-eastern region of Minas Gerais state, Brazil. A single stool sample was collected from individuals and eggs per gram of feces (epg) determined by the Kato-Katz fecal thick-smear technique [8] with two slides per patient. Individuals found to be monoinfected with hookworm were then enrolled into this study (*n* = 10). Blood was taken by venipuncture and the patients subsequently treated with a single dose of albendazole (400 mg) by the local health service personnel. Approximately 20 mL of blood was collected in heparinized tubes for separation of peripheral blood mononuclear cells (PBMCs). Nonendemic, egg-negative controls (*n* = 7) were recruited from the urban area of Belo Horizonte, Minas Gerais, which is considered an area of low to negligible transmission, especially outside the poor urban areas. Egg-negative controls consisted of volunteers from Centro de Pesquisas René Rachou who have not reported any intestinal helminth infection in the past. All volunteers provided written informed consent to participate in the study, and it was approved by the ethics committee from Centro de Pesquisas René Rachou-FIOCRUZ and the Brazilian “Conselho Nacional de Ética em Pesquisa” (CONEP).

### 2.2. Parasite ES Antigen Preparation

*Ancylostoma caninum *adult worms were recovered from the small intestines of stray dogs euthanized at the dog kennel of the Prefecture of Belo Horizonte during the regular leishmaniasis control program. Adult worm ES products (ES-AW) were obtained after incubation of the parasites for a period of 16–20 hours at 37°C in a humidified incubator and stored in aliquots at −70°C [9]. ES preparations from several days were pooled into 15 mL filtration tubes with a 5 kDa molecular weight cut-off filter (Millipore) and centrifuged for 1 hour at 4°C and 1,250 g. Antigen preparations were obtained after filtration in a 0.22 *μ*m low-protein binding syringe filter (Millipore) and the resulting protein concentration determined using the BCA protein assay kit (Pierce).

In order to obtain L_3_ for preparation of ES products, Harada-Mori fecal cultures from hookworm-infected individuals with more than 4,000 epg were performed [10]. Fifty-mili-liter plastic tubes was filled with 5 mL of tap water. Feces were distributed on filter paper strips on the upper two thirds of the filter paper, transferred to plastic tubes, and incubated in vertical position at 26–28°C for 7–10 days. The tubes were sealed with perforated Parafilm for air circulation. Fecal cultures were checked daily for water level, fungal contamination, and larvae in the water. After 7–10 days of incubation, the liquid was pooled in new tubes. To separate L_3_ from fecal material and fungi the tubes were thoroughly mixed on a vortex and left on the bench during 30 minutes for sedimentation. After carefully removing the supernatant the content of the tubes were merged and the pellet with L_3_ was resuspended with 40 mL of BU buffer at room temperature, as described by Hawdon et al. [11]. The solution was transferred to a small Baerman filter unit, containing several layers of gauze, and incubated for one hour at room temperature (RT). The resulting pellet of larvae was resuspended with BU buffer, mixed thoroughly, and the sedimentation step repeated. For bacterial decontamination, the pellet was incubated in BU/1% HCl buffer for 30 minutes at RT [11]. The larval suspension was transferred to a sterile 50 mL plastic tube and resuspension and sedimentation steps repeated under sterile conditions once with BU buffer, twice with minimal essential medium (Gibco), and once with RPMI-1640 medium (Gibco), containing 10% heat-inactivated normal human serum (ICN), 2% antibiotic-antimycotic solution (Sigma), and 1% L-glutamine (Winlab, Leicestershire, UK). After the last sedimentation step, the supernatant was removed, and the larvae resuspended in 5 mL complete RPMI-1640 medium (see above). For activation of larvae, 1 mL of larval suspension, containing approximately 10,000–20,000 larvae, was incubated in 3 mL of complete RPMI-1640 medium in 8-well cell multidish plates (NUNC) for 16–18 hours at 37°C and 5% CO_2_. Finally, the larval suspension was removed, pooled into 1.5 mL cups, and centrifuged at 20,800 g for 3 minutes. The supernatant was carefully removed and antigen preparations (ES-L_3_) from different larval cultures were pooled and stored at −70°C until used. Due to serum supplementation of cell culture medium for activation of L_3_, it was not possible to determine the specific protein concentration of larval ES products.

### 2.3. In Vitro Lymphocyte Proliferation

Separation of PBMC was performed as described elsewhere [12]. In 96-well cell culture plates, triplicates of 250,000 cells/well were added for antigen and mitogen stimulations in a final volume of 200 *μ*L of complete RPMI-1640 [12]. Final concentrations of stimulants determined to be optimal in cell culture were 35 *μ*g/mL for ES-AW antigens and 2.5 *μ*g/mL for phytohaemagglutinin (PHA)-L (Difco Laboratories, Detroit, MI, USA). For proliferation assays with ES-L_3_ antigen, a dose-response curve was performed at the beginning of the experiments, in which an increasing volume of L_3_ supernatant (5–50 *μ*L) was added to mitogen-stimulated PBMCs. 25 *μ*L of L_3_ supernatant resulted in a 56% inhibition of PBMC proliferation (data not shown) and was used in all subsequent experiments of proliferation and cytokine secretion. Cells were cultured at 37°C in a humidified 5% CO_2_ incubator. Tritiated thymidine (Amersham Pharmacia, São Paulo, Brazil; 0.5 *μ*Ci/culture; specific activity 6.7 Ci/mM) was added to the cultures at 48 h, and the cells were harvested 18 h later. Incorporated tritiated thymidine was determined in a liquid scintillation counter and the data expressed as stimulation indices (SIs) (mean proliferation of stimulated culture divided by mean proliferation of unstimulated culture). The stimulation index of PHA-stimulated lymphocytes served as reference value (100%), and percentages of costimulated cell cultures, either PHA plus ES-AW or PHA plus ES-L_3_, were calculated from this value.

### 2.4. Cytokine Detection in Cell Culture Supernatants

For production of cytokines and chemokines, 5 × 10^5^ PBMCs were cultivated in 48-well tissue culture plates (Costar, Corning, NY, USA) at a total volume of 400 *μ*L in complete RPMI-1640 for 2 days, using the same final mitogen and antigen concentrations as described above. Cell-free supernatants were stored at −70°C until cytokine quantification. Concentrations for IL-1*β*, IL-5, IL-6, IL-10, IFN-*γ*, and TNF-*α* were determined by enzyme-linked immunosorbent assay (ELISA) (R&D Systems, Minneapolis, USA). When necessary, samples were diluted with PBS in order to obtain a value within the range of the standard curve. ELISAs were performed in duplicate according to the manufacturer's protocols, using a total volume of 25 *μ*L per well in high-binding half-area plates (COSTAR, Corning, NY, USA). On each plate, serial dilutions of standards were run to construct standard curves with the following ranges of concentration: IL-1*β* (3.9–500 pg/mL); IL-5 (11.7–1,500 pg/mL); IL-6 (4.7–600 pg/mL); IL-10 (23.4–3,000 pg/mL); IFN-*γ* (7.8–1,000 pg/mL); TNF-*α* (7.8–1,000 pg/mL). The sensitivity for all ELISAs was lower than the last standard dilution. The colorimetric reaction was determined in an automated ELISA reader at 450 nm. Back calculations of cytokine concentrations from mean optical density values were interpolated from the standard curves by using a 4-parameter curve fitting program (SOFTmax PRO 3.1.2).

### 2.5. Cultivation of Lymphocytes and Living L_3_

For co-cultivation of PBMC with infective hookworm larvae, L_3_ from *Ancylostoma caninum*-infected dogs were obtained after fecal cultures (see above) and were a kind donation of Professor Walter dos Santos Lima and Professor Joziana Barçante (Federal University of Minas Gerais—UFMG). The *in vitro* cell cultures, 5 × 10^5^ PBMCs, were cultivated in 24-Transwell plates (pore size 3.0 *μ*m, COSTAR, Corning, NY, USA), at a total volume of 400 *μ*L in complete RPMI-1640. Cells were incubated, either in direct contact or separated by the plate insert, with approximately 20 L_3_ per well during 48 hours. Cell supernatants were obtained as described above and were stored at −70°C for future cytokine determination. Cytokine ELISAs were performed as described above.

### 2.6. Statistical Methods

For statistical evaluation, an SPSS 12.0 software package was used. Values from proliferation assays and cytokine concentrations were checked for normal distribution and were subsequently analysed by the nonparametric Mann-Whitney *U*-test for the comparison of two groups. Differences with a *P* value of less than  .05 were considered significant and are indicated in the text, table, or figure.

## 3. Results

### 3.1. Patients and Parasitological Exams

The mean age of hookworm-infected patients from Ladainha was higher than that in negative, nonendemic controls from Belo Horizonte (44.4 ± 13.8 years versus 29.4 ± 5.8 years); however this difference was not significant (*P* = .055). Also, nonendemic controls have been living in the urban area of Belo Horizonte. For the egg-negative group, helminth infections were not diagnosed at the time of the study and no past records of helminth infections were stated by the volunteers from the hypoendemic area. The geometric mean of hookworm egg counts was 789 epg (range: 11,892–684), with 7 patients harbouring light, 2 moderate, and 1 patient with heavy hookworm infections, according to the classification by the World Health Organization [13].

### 3.2. Lymphocyte Proliferation

**Figure 1:**
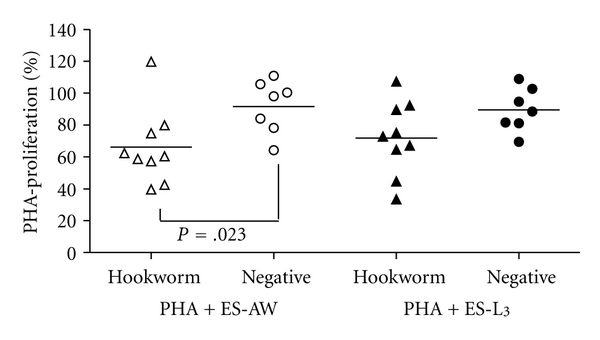
Inhibition of mitogen-stimulated PBMC proliferation after costimulation with adult worm ES (ES-AW, open symbols) and third-stage larval ES (ES-L_3_, full symbols) antigen in hookworm-infected (▲; *n* = 9) and egg-negative (●; *n* = 7) individuals. Symbols show individual percentages of proliferation in comparison with PHA-stimulated cultures only, and horizontal bars indicate mean values for each group. Significant differences between groups are indicated.

Figure 1 shows the individual percentages of PBMC proliferation after costimulation with PHA and hookworm ES-AW or ES-L_3_ in comparison to PHA-stimulated lymphocytes only. PBMCs from hookworm patients showed a lower percentage of proliferation when hookworm ES antigens were added to the lymphocyte cultures. After addition of ES-AW to PHA-stimulated lymphocytes the percentage of proliferation was significantly lower (*P* = .023) in hookworm-infected individuals. Notably, when PBMC cultures were stimulated with PHA only, no significant differences in the stimulation index resulted between the two patient groups (data not shown). 

### 3.3. Cytokine Secretion

Cytokine concentrations detected in cell culture supernatants are shown in Table 1. 

**Figure 2:**
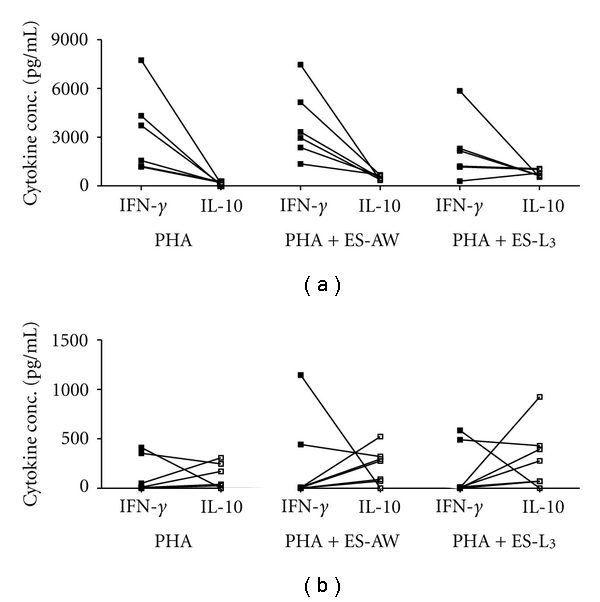
IFN-*γ* and IL-10 secretion in pg/mL in PBMC stimulated with PHA, PHA+ adult worm ES (ES-AW), or PHA+ ES-L_3_. (a) Paired IFN-*γ* and IL-10 cytokine concentrations in PBMCs from egg negative, nonendemic controls and (b) in hookworm-infected patients from an endemic area.

In unstimulated PBMC cultures from negative, nonendemic individuals lower concentrations of inflammatory IL-1*β*, IL-6, and TNF-*α* were detected when compared with participants with hookworm infection. For IL-1*β* and TNF-*α*, these differences were statistically significant (*P* < .01 and *P* < .05, resp.). When stimulated with PHA, PBMC from hookworm-infected individuals secreted significantly more IL-1*β* (*P* < .01) than egg-negative control subjects. Opposite to unstimulated control cultures, IL-6 and TNF-*α* secretions in PHA-stimulated or costimulated cultures were significantly lower in PBMCs from hookworm patients than in egg-negative individuals. For IL-5, low concentrations were detected in both, control and stimulated lymphocyte cultures from the two groups. However, unstimulated PBMCs from hookworm patients secreted significantly more IL-5 (*P* < .05) than PBMCs from egg-negative individuals. After addition of either ES-AW or ES-L_3_ antigen to PHA-stimulated cell cultures, individuals in both groups showed increased IL-10 secretion. However, this increase was significantly (*P* < .05) higher in the egg-negative controls. The IFN-*γ* secretion in participants with hookworm infection was significantly (*P* < .01) lower in all stimulated lymphocyte cultures when compared with egg-negative individuals.  Figure 2 shows the paired IFN-*γ* and IL-10 secretion for the two groups of patients separated in egg-negative controls (2A) and hookworm-infected individuals (2B). Upon stimulation, PBMC from egg-negative individuals secreted high levels of IFN-*γ* and considerably lower levels of IL-10. In contrast, PBMC from hookworm patients secreted equally low concentrations of IFN-*γ* and IL-10 (Figures 2(a) and 2(b)). 

### 3.4. Cultivation of Lymphocytes and Living L_3_

Table 2 shows the results obtained for cytokine secretion levels from PBMC cocultured with living L_3_ of *A. caninum*. Although the concentrations are lower if compared with the previous data, the differences in the secretion of inflammatory cytokines (IL-6, TNF-*α*) after co-cultivation with living L_3_ showed the same trend as described for soluble ES-L_3_ preparations from *N. americanus* (Table 1). These results show that this regulatory effect may be mediated by soluble factors secreted by the L_3_. On the other hand and in contrast with the soluble antigen preparations, co-cultivation of PBMC and L_3_ did not induce an increase in IL-10 secretion and cytokine concentrations remained low with no significant differences between the two groups (Table 2). IL-5 and IFN-*γ* secretion in PBMC cocultured with living L_3_ remained at marginal levels in both patient groups (data not shown). 

## 4. Discussion

Suppressed cellular responsiveness, either antigen-specific or polyclonal, and a skewed Th2 immune response are two of the hallmarks of helminth infections and have been extensively described for filarial infections [14, 15] and schistosomes [16–18]. In two independent studies, we have found reduced antigen-specific cellular responsiveness in hookworm patients in response to larval and adult worm soluble extracts [7, 19]. Interestingly, proliferative responses and cytokine secretion patterns were quite distinct for the different antigen preparations, with adult ES antigen inducing low type 1 and inflammatory responses especially in hookworm—infected individuals [7]. 

In the present paper, we have focused on the cellular response to ES antigenic preparations from two developmental stages of hookworms—infective third-stage larvae (L_3_) and adult worms (AWs). We were able to show that both ES preparations, L_3_ and AW, induce a considerable reduction in cell proliferative responses of polyclonally stimulated lymphocytes from hookworm-infected patients. The fact that we observe a reduction on polyclonal activation of PBMCs in infected participants with L_3_ ES antigen, suggests that down-modulatory mechanisms occur at the early L_3_ parasitic stage of infection in individuals previously sensitized with parasite antigens or that have a current infection. This observation is novel for hookworm infections and is certainly of major importance for vaccine development. In this context, induction of regulatory/suppressor responses induced by ES L_3_ antigens may have significant effect on the maintenance of parasite survival as well as on reinfection in endemic human populations. Importantly, Loukas et al. [20] showed that L_3_ and adult worms from *A. caninum* share ES and somatic antigens; these results together with the results presented in this paper suggest that common mechanisms of L_3_ and adult worms on the regulation of the immune response may facilitate parasite escape and survival. In contrast, it has also been shown by others that mechanisms of immunomodulation and the induction of pathological changes cannot be generalised between different hookworm species and that the induced inflammatory response in humans infected with *N. americanus* is much more subtle than, for example, after enteric *A. caninum* infections [21]. Also, in primary experimental infections in human volunteers and in hamsters it was shown that a state of reduced cellular responsiveness builds up slowly during the onset of patency, most probably with the more important contribution of the adult worm population that builds up with time [22, 23].

Due to the obvious difficulties in obtaining sufficient ES material from living human hookworm species, we used in our experiments adult worm ES products, as well as living larvae from *A. caninum* and compared the human cellular response to that of L_3_ ES products from *N. americanus*. We are well aware of the differences between human and dog hookworm infections [24] and that ES products from adult *A. caninum,* and *N. americanus* have been shown to have individual protein patterns and bind to distinct leukocyte populations [25] and different components might therefore have distinct effects on the human immune system [26]. However, the effects of total ES extracts, as described here, seem to be comparable for both dog and human hookworm ES product preparations. We are also aware of statistical limitations due to the low number of individuals in each group, which might impair to discover small differences between groups. However, we feel that in the present experimental setting with the unspecific polyclonal stimulation of lymphocytes by the mitogen PHA we are able to draw the presented conclusions, even if limited quantities of ES antigens forced us to use blood from a restricted number of individuals.

Experimental human infections monitored by capsule endoscopy have shown that adult worms still cause a significant degree of intestinal inflammation and newly arriving premature worms are expelled from the small intestine in the course of acute eosinophilic enteritis [27]. Nevertheless, adult hookworms have also been shown to resist intestinal inflammation and continued to parasitize the small intestine [28]. Furthermore, in repeatedly administered experimental human infections, even with as much as 250 L_3_, initial intestinal symptoms seem to vanish with every newly applied infection [29]. In our view, this indicates time- and dose-dependent mechanisms of hookworm immunosuppression with decreased intestinal inflammation, resulting in parasite persistence. There are already described mechanisms by which adult worm ES components induce suppression of the human immune response, for example, by the induction of apoptosis in reactive T cells, which avoids the infiltration of reactive host leukocytes to the place of adult worm attachment and facilitates worm survival [30], or by affecting dendritic cell maturation and differentiation of regulatory T cells [31].

We observed elevated levels of IL-1*β*, IL-6, and TNF-*α* concentrations in unstimulated PBMC cultures from infected individuals indicating that these volunteers have ongoing inflammatory processes. However, after *in vitro* PBMC stimulation infected patients showed a reduced capacity in secreting IL-6 and TNF-*α* either after PHA stimulation or in combination with ES antigen from both parasitic stages. Together with a low IFN-*γ* secretion in individuals with hookworm infection, our results point to a major role of ES products for reduced inflammatory/type 1 immune responses in the course of human hookworm infection. On the other hand, low to absent IL-5 secretion, either in response to ES preparations or in response to living parasites, respectively, suggests that ES antigens are poor inducers of type 2 immunity. This was also confirmed by previous antigen-specific stimulations of PBMC from hookworm-infected subjects [7], and similar results were described in PBMCs from nonexposed individuals which were incubated with live L_3_ from the filarial parasite *Brugia malayi* [32]. As other mechanism influencing the immunoregulation and the cytokine and chemokine milieu, it was reported that host eotaxin is specifically cleaved by metalloproteases from adult *N. americanus* ES secretions, which might be important in preventing recruitment and activation of eosinophils and even may influence Th2 responses [33]. Therefore, it would be interesting in future studies to collect additional information on IL-4 or IL-13 lymphocyte secretion patterns, or even on IL-21, a Th2 cytokine identified to be highly relevant in helminth infections [34]. Furthermore, during schistosome infection and migration through the skin, IL-10 seems to be a key regulator of the immune response [35]. However, in this study we observed a significantly lower IL-10 secretion in PBMC cultures from hookworm-infected patients when compared to negative individuals. On the other hand, a resulting immune response is the interplay between different and also counteracting cytokines. As such, the paired IFN-*γ* and IL-10 secretion for each patient upon stimulation of PBMC with ES antigens showed a completely different pattern between the two patient groups; for example, upon stimulation there is a relatively higher IFN-*γ* than IL-10 level in egg-negative controls and equally low levels of IFN-*γ* and IL-10 in hookworm-infected individuals. In our view, this ES antigen-induced low IFN-*γ*/IL-10 ratio in hookworm-infected patients may contribute to the enhanced suppression of the immune response, with reduced cellular reactivity, and a diminished inflammatory response. Interestingly, an elegant study in *Heligmosomoides polygyrus*-infected mice has recently shown that adult worm ES products from this rodent nematode inhibit the maturation and function of dendritic cells and may drive the differentiation of IL-10 producing T_reg_ cells. As a result, T cell, cytokine, and antibody responses are suppressed in a generalized manner [36]. The putative role of an altered phenotype and function of dendritic cells in hookworm-infected patients on the observed suppression/regulation of the immune response in these patients has recently been published by our group [37]. Also, the important contribution of alternatively activated macrophages in the course of intestinal helminth infections has recently been reviewed and emphasized by Kreider et al. [38] and might be important in directing the immune response during human hookworm infections. As such, the effect of hookworm ES products on antigen presenting cells, as well as the induction of regulatory T cell cytokines, such as TGF-*β* or IL-17, deserves further investigation.

In summary, we were able to show that hookworm ES products from *A. caninum* adult worms or L_3_ induce immune mechanisms that significantly reduce proliferative responses in mitogen-activated PBMC from hookworm-infected individuals. Furthermore, a significant downmodulation of inflammatory cytokine secretion, as well as a lower IFN-*γ*/IL-10 ratio, resulted in stimulated PBMC from hookworm patients, when compared to nonendemic, egg-negative controls, factors that might be decisive for early larval survival or adult worm persistence in the gut.

## Figures and Tables

**Table 1:** Cytokine concentrations (pg/mL) in supernatants from PHA-stimulated PBMC cultures with or without costimulation by adult worm ES (ES-AW) or third-stage larval ES (ES-L_3_) antigen. Values from hookworm-infected individuals (*n* = 7) and negative controls (*n* = 6) are compared and indicated as mean values ± standard errors. Significant differences between groups are indicated with an asterisk (*P < .01;  **P < .05).

	Group	Control	PHA	PHA+ES-AW	PHA+ES-L_3_
IL-1*β*	Hookworm	1,034 ± 239*	1,446 ± 176*	1,421 ± 265	1,456 ± 320
	Negative controls	27 ± 27*	215 ± 114*	1,562 ± 424	785 ± 340
IL-6	Hookworm	52,382 ± 14,323	26,553 ± 12,000**	27,865 ± 7,464*	22,056 ± 4,763*
	Negative controls	18,452 ± 4,400	63,409 ± 14,530**	102,499 ± 11,146*	87,143 ± 14,244*
TNF-*α*	Hookworm	3,630 ± 2,621**	297 ± 185**	741 ± 300*	885 ± 333**
	Negative controls	306 ± 114**	2,104 ± 538**	5,638 ± 1,385*	2,820 ± 832**
IL-5	Hookworm	35 ± 19**	40 ± 20	12 ± 7	53 ± 40
	Negative controls	5 ± 5**	45 ± 22	7 ± 6	37 ± 17
IL-10	Hookworm	63 ± 26	113 ± 48	225 ± 69**	310 ± 120**
	Negative controls	0	157 ± 33	526 ± 58**	776 ± 89**
IFN-*γ*	Hookworm	43 ± 22	119 ± 69*	230 ± 165*	158 ± 99*
	Negative controls	0	3,288 ± 1,046*	3770 ± 899*	2,168 ± 796*

**Table 2:** Cytokine concentrations (pg/mL) in supernatants of PBMC cultures cocultured with living L_3_ from *Ancylostoma caninum*. Cells were either separated from the L_3_ by a membrane insert (cells/L_3_) or were cultivated in direct contact with the parasites (cells+L_3_). Values from hookworm-infected individuals (*n* = 7) and negative controls (*n* = 6) are compared and indicated as mean values ± standard errors. Significant differences between patient groups are indicated with an asterisk (*P < .01;   **P < .05).

	Group	Control	Cells/L_3_	Cells+L_3_
IL-1*β*	Hookworm	1,034 ± 239*	833 ± 149	1,161 ± 218
	Negative controls	27 ± 27*	424 ± 134	1,470 ± 494
IL-6	Hookworm	52,382 ± 14,323	8,703 ± 2,258*	12,124 ± 2,548*
	Negative controls	18,452 ± 4,400	62,750 ± 18,488*	59,185 ± 15,443*
TNF-*α*	Hookworm	3,630 ± 2,621**	351 ± 110*	404 ± 94*
	Negative controls	306 ± 114**	1,784 ± 362*	2,163 ± 221*
IL-10	Hookworm	63 ± 26	86 ± 48	37 ± 26
	Negative controls	0	18 ± 16	92 ± 31
